# Long-term evaluation of the safety and efficacy of recombinant human pentraxin-2 (rhPTX-2) in patients with idiopathic pulmonary fibrosis (IPF): an open-label extension study

**DOI:** 10.1186/s12931-022-02047-0

**Published:** 2022-05-21

**Authors:** Ganesh Raghu, Mark J. Hamblin, A. Whitney Brown, Jeffrey A. Golden, Lawrence A. Ho, Marlies S. Wijsenbeek, Martina Vasakova, Alberto Pesci, Danielle E. Antin-Ozerkis, Keith C. Meyer, Michael Kreuter, Tracy Burgess, Nikhil Kamath, Francis Donaldson, Luca Richeldi

**Affiliations:** 1 Center for Interstitial Lung Diseases, Department of Medicine and Laboratory Medicine, University of Washington, Seattle, WA USA; 2 Pulmonary and Critical Care Medicine, University of Kansas Medical Center, Kansas City, KS USA; 3 Inova Advanced Lung Disease and Transplant Program, Inova Fairfax Hospital, Falls Church, VA USA; 4 Department of Medicine, University of California, San Francisco, San Francisco, CA USA; 5 Department of Respiratory Medicine, Erasmus MC, University Medical Center, Rotterdam, Netherlands; 6 Department of Respiratory Medicine, First Faculty of Medicine of Charles University and Thomayer Hospital, Prague, Czech Republic; 7 School of Medicine and Surgery, University of Milano-Bicocca, ASST-Monza, Milano, Italy; 8 Pulmonary, Critical Care, and Sleep Medicine, Yale School of Medicine, New Haven, CT USA; 9 Department of Medicine, Division of Pulmonary and Critical Care, University of Wisconsin School of Medicine and Public Health, Madison, WI USA; 10 Center for Interstitial and Rare Lung Diseases, Department of Pneumology, Thoraxklinik, University of Heidelberg and German Center for Lung Research, Heidelberg, Germany; 11 Genentech, Inc., South San Francisco, CA USA; 12 Roche Products Ltd., Welwyn Garden City, UK; 13 Fondazione Policlinico Universitario A Gemelli IRCCS, Università Cattolica del Sacro Cuore, Rome, Italy

**Keywords:** Recombinant human pentraxin-2 (rhPTX-2), Idiopathic pulmonary fibrosis (IPF), Long-term, Safety, Open-label extension, Forced vital capacity (FVC), 6-minute walk distance (6MWD)

## Abstract

**Background:**

Recombinant human pentraxin-2 (rhPTX-2) significantly decreased decline in percent predicted forced vital capacity (FVC) and stabilized 6-min walk distance (6MWD) in patients with idiopathic pulmonary fibrosis (IPF) during the 28-week, placebo-controlled, randomized period of the Phase II PRM-151–202 study. Interim (76-week) data from the open-label extension (OLE) demonstrated sustained safety and efficacy with rhPTX-2 treatment. Here, we present the entire long-term OLE safety and efficacy data to 128 weeks.

**Methods:**

Patients who completed the randomized PRM-151–202 study period were eligible for the OLE, during which all patients received rhPTX-2, having started rhPTX-2 (i.e., crossed from placebo) or continued rhPTX-2 after Week 28. rhPTX-2 was administered in 28-week cycles, with 10 mg/kg intravenous infusions (60 min) on Days 1, 3, and 5 in the first week of each cycle, then one infusion every 4 weeks up to Week 128. The OLE primary objective was to assess the long-term safety and tolerability of rhPTX-2. Other outcomes included FVC, 6MWD, and patient-reported outcomes (descriptive analysis).

**Results:**

All 111 patients who completed the randomized period entered the OLE (n = 37 started rhPTX-2; n = 74 continued rhPTX-2); 57 (51.4%) completed to Week 128. The treatment-emergent adverse event (TEAE) profile was consistent with the randomized period, with the majority of TEAEs graded mild or moderate. Serious TEAEs occurred in 47 patients (42.3%), most frequently IPF (n = 11; 9.9%), pneumonia (n = 7; 6.3%), and acute respiratory failure (n = 3; 2.7%). Three patients underwent lung transplantation. Most serious TEAEs (and all 14 fatal events) were considered unrelated to rhPTX-2 treatment. For patients starting vs continuing rhPTX-2, mean (95% confidence interval) changes from baseline to Week 128 were, respectively, − 6.2% (− 7.7; − 4.6) and − 5.7% (− 8.0; − 3.3) for percent predicted FVC and − 36.3 m (− 65.8; − 6.9) and − 28.9 m (− 54.3; − 3.6) for 6MWD; however, conclusions were limited by patient numbers at Week 128.

**Conclusions:**

Long-term treatment (up to 128 weeks) with rhPTX-2 was well tolerated in patients with IPF, with no new safety signals emerging in the OLE. The limited efficacy data over 128 weeks may suggest a trend towards a treatment effect.

*Trial registration* NCT02550873; EudraCT 2014-004782-24.

## Introduction

Idiopathic pulmonary fibrosis (IPF) is a chronic interstitial lung disease that is characterized by pathologic fibroproliferative healing of repeated alveolar injury in disposed individuals usually of older age [1, 2]. The progressive fibrosis in IPF distorts the lung architecture and results in irreversible loss of lung function, diminished functional capacity, loss of quality of life, and a poor prognosis [1–3].

Current treatments approved for IPF include two antifibrotics; pirfenidone and nintedanib [4–7]. Pirfenidone and nintedanib may improve the life expectancy of patients with IPF [8–10]. Although these drugs are able to slow down disease progression, they cannot stop it completely [11, 12]. To date, there is no therapy that can reverse the fibrosis in IPF and restore the lung parenchyma to normal. Also, many patients experience side effects with pirfenidone and nintedanib. Thus, there is an unmet need for therapies that are better tolerated and can further slow or halt IPF disease progression.

Recombinant human pentraxin-2 (rhPTX-2; previously known as PRM-151) is a recombinant form of a naturally occurring protein called pentraxin-2, which is being investigated as a possible treatment for IPF. Results from the Phase II, randomized, double-blind, placebo-controlled portion of the PRM-151-202 study investigating rhPTX-2 in patients with IPF (ClinicalTrials.gov: NCT02550873) have been published [13]. The placebo-controlled period of PRM-151-202 showed that rhPTX-2 was able to significantly decrease the decline in percent predicted forced vital capacity (FVC) and stabilize 6-min walk distance (6MWD) after 28 weeks of treatment, compared with placebo [13]. Efficacy trends of rhPTX-2 were observed within the subgroups of patients who were receiving either pirfenidone or nintedanib concomitantly, as well as patients who were receiving rhPTX-2 as monotherapy [13]. The observed effect of rhPTX-2 on 6MWD within this Phase II study was novel, being the first clinical trial in IPF to have shown stabilization of patients’ functional status, as defined by 6MWD [13, 14].

Patients who completed the randomized period of the Phase II trial without rapid disease progression or study treatment discontinuation due to toxicity were eligible to participate in the open-label extension (OLE) period, during which all patients were treated with rhPTX-2. Patients initially in the placebo arm of the study crossed over to receive rhPTX-2, and patients who were in the treatment arm continued rhPTX-2 therapy. An interim analysis, up to Week 76 of the OLE, has been previously published, and supported findings seen in the randomized phase of the study with regard to both the safety and efficacy of rhPTX-2 [13, 15]. The effects on percent predicted FVC and 6MWD were persistent for those who continued on treatment with rhPTX-2, and patients who crossed over from the placebo arm also appeared to derive efficacy from rhPTX-2 treatment [15].

Here, we present the final long-term results from the OLE period of the PRM-151-202 study, which aimed to assess the longer-term safety and efficacy of rhPTX-2 in patients with IPF from baseline to Week 128.

## Methods

### Study design and participants

The Phase II study, PRM-151-202, was composed of a randomized, double-blind, placebo-controlled period of 28 weeks and an OLE period lasting up to Week 128 (ClinicalTrials.gov: NCT02550873). The trial design and primary outcomes of the Phase II trial and OLE results up to Week 76 have been described previously [13, 15].

Eligible patients were aged 40–80 years with a diagnosis of IPF according to the 2011 American Thoracic Society/European Respiratory Society/Japanese Respiratory Society/Latin American Thoracic Association criteria [13, 15, 16]. At the start of the study, patients were required to have: percent predicted FVC ≥ 50% to ≤ 90%; percent predicted carbon monoxide diffusing capacity (DLco) ≥ 25% to ≤ 90%; minimum 6MWD of 150 m; and forced expiratory volume in 1 s (FEV_1_)/FVC ratio greater than 0.7.

Study participants were randomized in a 2:1 ratio to receive 10 mg/kg rhPTX-2 or placebo by intravenous (IV) infusion on Days 1, 3, and 5, followed by one IV infusion every 4 weeks for 24 weeks during the randomized period. There was an End-of-Study follow-up assessment at Week 28. Patients who participated in the OLE received 10 mg/kg rhPTX-2 as a loading dose IV infusion on Days 1, 3, and 5, followed by one IV infusion every 4 weeks. Administration of a loading dose of rhPTX-2 on Days 1, 3, and 5 was repeated every 28 weeks up to Week 128. Patients not on background therapy of nintedanib or pirfenidone could begin or restart treatment with pirfenidone or nintedanib after the Week 28 End-of-Study assessment, if desired.

This trial was conducted in accordance with the principles of the Declaration of Helsinki and Good Clinical Practice guidelines. Written informed consent was obtained from each patient or caregiver as appropriate before screening, except at two sites where consent was attained separately at the start of the Phase II trial and again at the start of the OLE period.

### Safety and efficacy assessments

The primary objective of the OLE was to assess the long-term safety and tolerability of rhPTX-2. Safety was determined by analyzing adverse events (AEs) that emerged during the OLE period up to Week 128.

Efficacy was assessed by exploratory analysis of FVC, 6MWD, and DLco up to Week 128. Patient-reported outcomes were the King’s Brief Interstitial Lung Disease questionnaire (K-BILD) and the Leicester Cough Questionnaire (LCQ). In addition, as part of the laboratory evaluations, anti-pentraxin-2 antibody titers were evaluated up to Week 128.

### Statistical analysis

Five patient groups were assessed and defined as follows:

- Randomized: all patients who were randomized.
- All Treated Set (ATS): all randomized patients who received at least one dose of study drug during the randomized period, analyzed as randomized, regardless of actual treatment received.
- Safety Analysis Set (SAF): all randomized patients who received at least one dose of study drug during the randomized period, analyzed as treated.
- ATS and the SAF of the OLE (ATS-OLE; SAF-OLE); both defined as all randomized patients who received at least one dose of rhPTX-2 during the OLE.

Safety analyses were performed for all AEs (documented according to Medical Dictionary for Regulatory Activities, version 19.0) and medications that started after patients had received at least one dose of rhPTX-2. Events or medications that started during or after the first dose of the OLE were considered treatment-emergent for the OLE period. Incidence of AEs and time to study discontinuation (for any reason) were summarized descriptively in the SAF and SAF-OLE populations, as well as the following parameters: time to first AE of cough; incidence and time to first respiratory decline event; incidence and time to first exacerbation of IPF; time to lung transplantation (if applicable); and time to death.

Efficacy analyses were conducted from baseline until Week 128 and were summarized descriptively for: percent predicted FVC and 6MWD (every 4 weeks to Week 52 and then every 12 weeks); DLco (at baseline, Week 28, Week 76, and Week 128); K-BILD and LCQ (every 4 weeks to Week 52 and then every 12 weeks). For each efficacy parameter, descriptive statistics for raw values and change from baseline at each time point were computed by initial randomized group for the ATS and ATS-OLE up to Week 128. No sensitivity analyses were performed. Anti-pentraxin-2 antibody titers were evaluated every 12 weeks, with descriptive statistics computed for the ATS and ATS-OLE to Week 128.

## Results

Overall, 111 of 117 patients (94.9%) randomized in the Phase II study completed the randomized period. All of these patients participated in the OLE; 37/39 patients (94.9%) who received placebo during the randomized period started rhPTX-2 in the OLE (hereafter referred to as ‘patients who started rhPTX-2’), and 74/78 patients (94.9%) who received rhPTX-2 continued after completion of the randomized period. Patient enrollment to the Phase II study began in September 2015, with the final OLE visit in April 2019.

### Patient demographics

Patient demographics have previously been reported [15]. Briefly, 80.2% (n = 89) of patients enrolled in the OLE were male and the mean (standard deviation [SD]) age of patients was 68.6 (6.5) years. In the overall cohort, the mean (SD) time since diagnosis of IPF was 3.8 (2.3) years and the median (range) was 3.0 (1–13) years; in the subgroups of patients who started rhPTX-2 and continued rhPTX-2, the median time since diagnosis of IPF was 3.0 years for both.

**Fig. 1:**
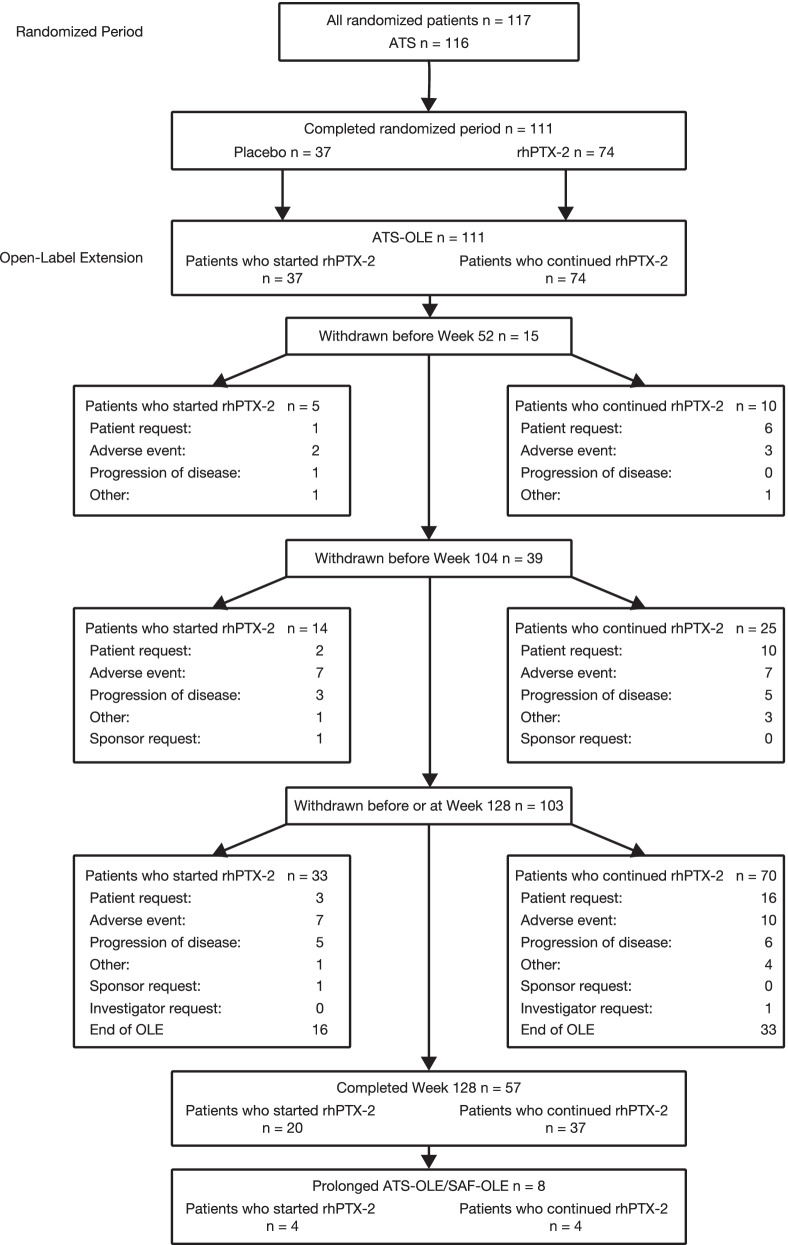
Patient disposition**.** ATS, All Treated Set; ATS-OLE, All Treated Set of the open-label extension; OLE, open-label extension; rhPTX-2, recombinant human pentraxin-2; SAF-OLE, Safety Analysis Set of the open-label extension

The disposition of patients who participated in the OLE is provided in Fig. 1. Overall, 51.4% (n = 57) of patients enrolled in the OLE completed 128 weeks; most of these patients (n = 49/57) discontinued the study at the Week 128 visit. In total, 103 of 111 patients (92.8%) enrolled in the OLE discontinued the study at or before the Week 128 visit; this included 52.4% (n = 54/103) who discontinued prior to completion of 128 weeks and 47.6% (n = 49/103) who completed the OLE at Week 128. Reasons for early study discontinuation were patient’s request (n = 19; 18.4%), AEs (n = 17; 16.5%), progression of disease (n = 11; 10.7%), other (n = 5; 4.9%), investigator request (n = 1; 1.0%), and sponsor request (n = 1; 1.0%). Of the 19 patients who discontinued due to ‘patient request’, 18 patients provided specific reasons for withdrawal, and the main reasons included travel issues (n = 6), the patient wanted to pursue a lung transplant (n = 4), and withdrawal of consent (n = 3). It should be noted that none of these patients reported safety, tolerability, or quality-of-life issues as a reason for discontinuing. The remaining 8 of 111 patients enrolled (7.2%) continued rhPTX-2 treatment past the end of the OLE; a single site (the first site to enroll patients in PRM-151-202) had been permitted to continue treatment beyond 128 weeks due to the site’s interpretation of the protocol (having been granted a request to the sponsor to allow participation in the study beyond 128 weeks). The last patient’s final visit at that site was set to coincide with the final Week 128 visit of the last patient from the other 17 sites.

The mean duration of exposure to rhPTX-2 was 505 days for patients who started rhPTX-2 and 705 days for patients who continued rhPTX-2. At the start of the OLE, 75.7% (n = 84/111) of patients were receiving background therapy (either pirfenidone or nintedanib). At Week 128, 82.5% (n = 47/57) of patients were receiving background therapy, with an even distribution between patients who started rhPTX-2 (n = 16/20; 80%) and patients who continued rhPTX-2 (n = 31/37; 83.8%). 

### Safety endpoints

Overall, 94.6% (n = 105) of patients in the SAF-OLE population experienced 1052 treatment-emergent AEs (TEAEs) during the OLE, up to and including Week 128 (Table 1). The most frequently reported TEAEs were IPF (29.7%), dyspnea (28.8%), cough (27.0%), and upper respiratory tract infection (23.4%) (Table 2). Differences of ≥ 10% between patients who started rhPTX-2 and patients who continued rhPTX-2 were seen for dyspnea (16.2% vs 35.1%), cough (37.8% vs 21.6%), diarrhea (5.4% vs 20.3%), and bronchitis (21.6% vs 10.8%) (Table 2).

**Table 1:** Summary of TEAEs emerging during the OLE up to Week 128 (SAF-OLE population)

Event, n (%)	Patients who started rhPTX-2 (n = 37)	Patients who continued rhPTX-2 (n = 74)	Total (n = 111)
Any TEAE	36 (97.3)	69 (93.2)	105 (94.6)
Any TEAE leading to permanent study drug discontinuation	10 (27.0)	18 (24.3)	28 (25.2)
Any TEAE leading to hospitalization	13 (35.1)	25 (33.8)	38 (34.2)
Any mild TEAE	31 (83.8)	65 (87.8)	96 (86.5)
Any moderate TEAE	28 (75.7)	53 (71.6)	81 (73.0)
Any severe TEAE	8 (21.6)	23 (31.1)	31 (27.9)
Any life-threatening TEAE	2 (5.4)	1 (1.4)	3 (2.7)
Any TEAE possibly or probably related to study drug	14 (37.8)	24 (32.4)	38 (34.2)
Any IRR TEAE	3 (8.1)	7 (9.5)	10 (9.0)
Any respiratory decline TEAE	8 (21.6)	31 (41.9)	39 (35.1)
Any IPF exacerbations reported as TEAEs	1 (2.7)	6 (8.1)	7 (6.3)
Any serious TEAE	15 (40.5)	32 (43.2)	47 (42.3)
Any life-threatening serious TEAE	2 (5.4)	1 (1.4)	3 (2.7)
Death	4 (10.8)	10 (13.5)	14 (12.6)
Any serious TEAE possibly or probably related to study drug	1 (2.7)	1 (1.4)	2 (1.8)
Any IRR serious TEAE	0	1 (1.4)	1 (0.9)
Any respiratory decline serious TEAE	6 (16.2)	16 (21.6)	22 (19.8)

IPF, idiopathic pulmonary fibrosis; IRR, infusion-related reaction; OLE, open-label extension; rhPTX-2, recombinant human pentraxin-2; SAF-OLE, Safety Analysis Set of the open-label extension; TEAE, treatment-emergent adverse event

TEAEs were documented according to Medical Dictionary for Regulatory Activities, version 19.0

**Table 2:** TEAEs that occurred in > 5% of patients overall during the OLE up to and including Week 128 (SAF-OLE population)

Preferred term, n (%)	Patients who started rhPTX-2 (n = 37)	Patients who continued rhPTX-2 (n = 74)	Total (n = 111)
Idiopathic pulmonary fibrosis	11 (29.7)	22 (29.7)	33 (29.7)
Dyspnea	6 (16.2)	26 (35.1)	32 (28.8)
Cough	14 (37.8)	16 (21.6)	30 (27.0)
Upper respiratory tract infection	8 (21.6)	18 (24.3)	26 (23.4)
Fatigue	5 (13.5)	14 (18.9)	19 (17.1)
Nasopharyngitis	5 (13.5)	13 (17.6)	18 (16.2)
Diarrhea	2 (5.4)	15 (20.3)	17 (15.3)
Bronchitis	8 (21.6)	8 (10.8)	16 (14.4)
Hypoxia	5 (13.5)	11 (14.9)	16 (14.4)
Arthralgia	4 (10.8)	8 (10.8)	12 (10.8)
Dizziness	2 (5.4)	10 (13.5)	12 (10.8)
Pneumonia	3 (8.1)	9 (12.2)	12 (10.8)
Back pain	3 (8.1)	8 (10.8)	11 (9.9)
Respiratory tract infection	2 (5.4)	9 (12.2)	11 (9.9)
Productive cough	1 (2.7)	9 (12.2)	10 (9.0)
Pulmonary hypertension	4 (10.8)	6 (8.1)	10 (9.0)
Dyspnea exertional	1 (2.7)	8 (10.8)	9 (8.1)
Hypertension	2 (5.4)	6 (8.1)	8 (7.2)
Rash	1 (2.7)	7 (9.5)	8 (7.2)
Blood pressure fluctuation	3 (8.1)	4 (5.4)	7 (6.3)
Gastroesophageal reflux disease	2 (5.4)	5 (6.8)	7 (6.3)
Nausea	1 (2.7)	6 (8.1)	7 (6.3)
Peripheral edema	4 (10.8)	3 (4.1)	7 (6.3)
Headache	1 (2.7)	5 (6.8)	6 (5.4)
Influenza	2 (5.4)	4 (5.4)	6 (5.4)
Pain in extremity	2 (5.4)	4 (5.4)	6 (5.4)
Pyrexia	3 (8.1)	3 (4.1)	6 (5.4)
Sinusitis	3 (8.1)	3 (4.1)	6 (5.4)

OLE, open-label extension; rhPTX-2, recombinant human pentraxin-2; SAF-OLE, Safety Analysis Set of the open-label extension; TEAE, treatment-emergent adverse event

TEAEs were documented according to Medical Dictionary for Regulatory Activities, version 19.0

As summarized in Table 1, 34.2% (n = 38) of patients reported events considered possibly or probably related to the study drug, most commonly: blood pressure fluctuation (8.1% of patients who started rhPTX-2 and 5.4% of patients who continued rhPTX-2); fatigue (2.7% and 6.8%, respectively); cough (8.1% and 1.4%, respectively); diarrhea (0% and 4.1%, respectively); and hypertension (2.7% in each group). TEAEs led to permanent discontinuation of study drug in 25.2% (n = 28) of patients (27.0% of patients who started rhPTX-2 and 24.3% of patients who continued rhPTX-2), most frequently IPF (n = 10; 9.0%). Serious TEAEs were reported for 42.3% (n = 47) of patients, most frequently IPF (n = 11; 9.9%), pneumonia (n = 7; 6.3%), and acute respiratory failure (n = 3; 2.7%). One patient who continued rhPTX-2 experienced three serious TEAEs (two events of tendonitis interfering with ambulation and one event of dysgeusia leading to weight loss) that were considered possibly or probably related to the study drug. Severe TEAEs were reported in 27.9% (n = 31) of patients, and life-threatening events in 2.7% of patients (n = 3; acute respiratory failure, pneumonia, small cell lung cancer, n = 1 each; not deemed to be related to study treatment). Fatal TEAEs were reported in 12.6% (n = 14) of patients, most common of which were IPF (8.1% [n = 3] of patients who started rhPTX-2 and 5.4% [n = 4] of patients who continued rhPTX-2), followed by myocardial infarction and pneumonia (each 2.7% [n = 2] of patients who continued rhPTX-2). One patient had three reported events that occurred on the day of death and thus all three events were registered as fatal (IPF exacerbation, acute respiratory failure secondary to IPF progression, and hypotension). None of the fatal TEAEs were considered related to study drug.

Three patients underwent lung transplantation during the OLE: one patient who started rhPTX-2 (time to transplant: 326 days) and two patients who continued rhPTX-2 (time to transplant: 225 and 746 days; Table 3). Time to first respiratory decline event, first exacerbation of IPF, first cough AE, study discontinuation, and death are also presented in Table 3.

**Table 3:** Time to event analysis during the OLE up to and including Week 128 (SAF-OLE population)

Event, patients with recorded event (n), median days (range)	Patients who started rhPTX-2 (n = 37)	Patients who continued rhPTX-2 (n = 74)	Total (n = 111)
Time to first respiratory decline event	n = 9	n = 39	n = 48
	372.0 (47.0–808.0)	374.0 (5.0–832.0)	373.0 (5.0–832.0)
Time to first exacerbation of IPF	n = 1	n = 7	n = 8
	423.0 (423.0–423.0)	397.0 (5.0–740.0)	410.0 (5.0–740.0)
Time to first AE of cough	n = 17	n = 28	n = 45
	303.0 (56.0–823.0)	140.5 (1.0–786.0)	205.0 (1.0–823.0)
Time to lung transplantation	n = 1	n = 2	n = 3
	326.0 (326.0–326.0)	485.5 (225.0–746.0)	326.0 (225.0–746.0)
Time to discontinuation of study drug	n = 17	n = 35	n = 52
	478.0 (201.0–810.0)	506.0 (199.0–841.0)	479.0 (199.0–841.0)
Time to death	n = 4	n = 10	n = 14
	525.5 (206.0–858.0)	488.5 (342.0–784.0)	488.5 (206.0–858.0)

AE, adverse event; IPF, idiopathic pulmonary fibrosis; OLE, open-label extension; rhPTX-2, recombinant human pentraxin-2; SAF-OLE, Safety Analysis Set of the open-label extension

TEAEs were documented according to Medical Dictionary for Regulatory Activities, version 19.0

#### Infusion-related reactions

Ten patients (9.0%) experienced infusion-related reactions (IRRs) during the OLE (Table 1); the most frequently reported IRR was blood pressure fluctuation in six patients (5.4%), and hypertensive crisis, pallor, tendonitis, dizziness, and IRR were reported in one patient each (0.9%). Two IRR events (tendonitis) in one patient were reported as serious, but no IRR was fatal or life-threatening, and the majority of events were mild to moderate and did not require discontinuation of study treatment. No anti-drug antibodies (ADAs) were detectable in these patients (see below).

#### Anti-drug antibodies

At baseline, no patients in the ATS-OLE population had ADAs (defined as a sample that was immunoreactive, irrespective of the antibody titer). Post-baseline, 20 patients (17.5%) had at least one ADA-positive assay; nine patients who started rhPTX-2 (24.3%) and 11 patients who continued rhPTX-2 (14.9%). In the SAF-OLE population, the majority of TEAEs in each category occurred in the absence of ADAs, and no ADAs were detected in patients with IRRs. In the presence of ADAs, 18 patients (16.2%) experienced TEAEs, and these were considered related to study drug in four patients (3.6%: pulmonary pain, hypoxia, arrhythmia, bronchitis, and International Normalized Ratio increased [n = 1]; liver disorder [n = 1]; cough and productive cough [n = 1]; chills [n = 1]).

### Efficacy endpoints

**Fig. 2:**
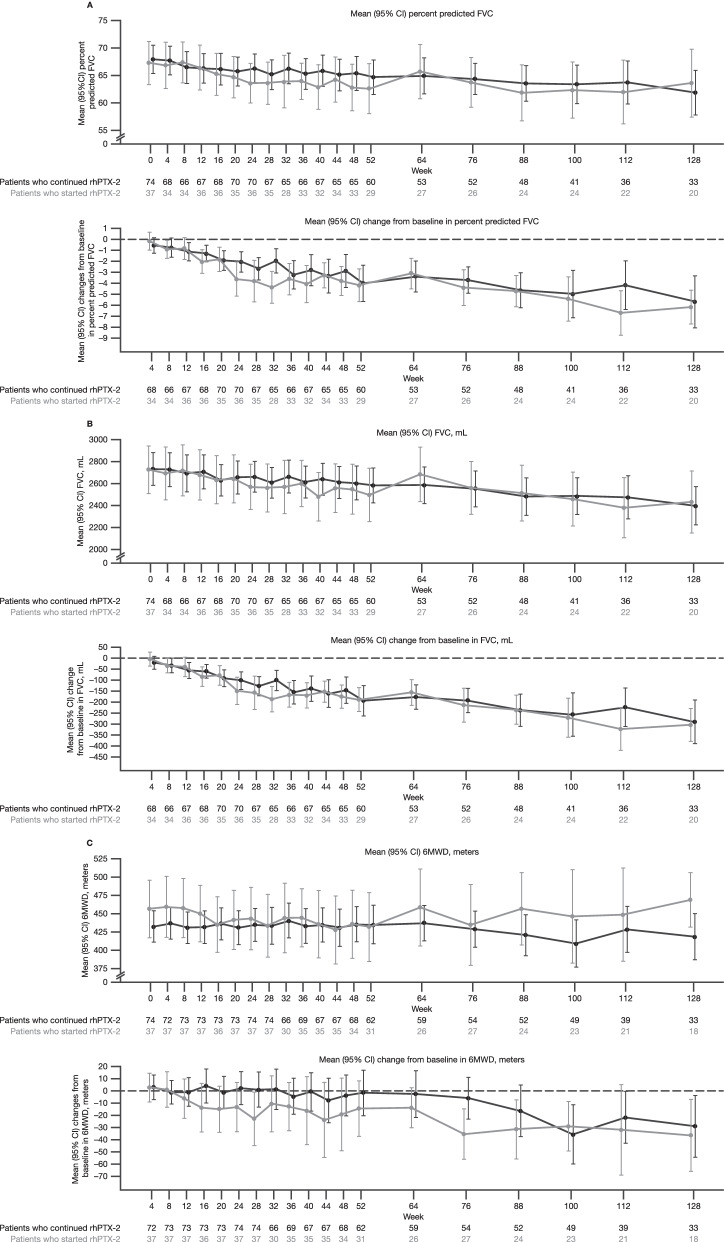
Mean and mean change from baseline in: **A** percent predicted FVC; **B** absolute FVC; **C** 6MWD, over 128 weeks (ATS-OLE population)**.** 6MWD, 6-min walk distance; ATS-OLE, All Treated Set of the open-label extension; CI, confidence interval; FVC, forced vital capacity; rhPTX-2, recombinant human pentraxin-2

The mean (95% confidence interval [CI]) raw values of percent predicted FVC at baseline, Week 28, and Week 128 were 67.2% (63.4; 71.1), 63.6% (59.8; 67.4), and 63.6% (57.4; 69.8), respectively, for patients who started rhPTX-2 from Week 28, and 67.9% (65.3; 70.4), 65.1% (62.4; 67.8), and 61.9% (57.8; 65.9), respectively, for patients who continued rhPTX-2 from Week 28. There was no difference in the mean (95% CI) change from baseline to Week 128 of percent predicted FVC between patients who started rhPTX-2 (− 6.2% [− 7.7; − 4.6]) and patients who continued rhPTX-2 (− 5.7% [− 8.0; − 3.3]). The mean raw values and changes from baseline for percent predicted FVC over time to Week 128 are presented graphically in Fig. 2A. The mean raw values and changes from baseline for decline in absolute FVC (mL) over time are presented in Fig. 2B.

The mean raw values and changes from baseline for decline in 6MWD over time are presented in Fig. 2C. The mean (95% CI) change from baseline to Week 128 in 6MWD was − 36.3 m (− 65.8; − 6.9) for patients who started rhPTX − 2 and − 28.9 m (− 54.3; − 3.6) for those who continued rhPTX-2.

The mean (SD) changes in percent predicted DLco from baseline to Week 128 were − 6.7% (6.0) and − 7.1% (8.5) for patients who started rhPTX-2 and patients who continued rhPTX-2, respectively. There was no difference between the two groups in the change in mean (SD) K-BILD total score at Week 128 (–9.9 [12.3] for patients who started rhPTX-2 compared with − 8.5 [16.4] for patients who continued rhPTX-2). Mean (SD) K-BILD total score at baseline of the OLE was 77.8 (18.4) for patients who started rhPTX-2 compared with 71.8 (18.8) for patients who continued rhPTX-2, and 76.9 (19.5) and 70.2 (17.1) at Week 128, respectively. There was no change in LCQ total score over time. At Week 128, the mean (SD) decrease in LCQ total score was − 1.1 (2.2) for patients who started rhPTX-2 compared with − 1.5 (4.0) for patients who continued rhPTX-2.

Any correlation between outcomes and the different rhPTX-2 and licensed antifibrotic combinations is unknown due to the limited number of patients.

## Discussion

PRM-151-202 was a Phase II study designed to investigate the efficacy and safety of rhPTX-2 in patients with IPF. The study consisted of a randomized, placebo-controlled, double-blind treatment period up to Week 28, followed by an OLE period up to Week 128. Here, we present results from the entire OLE period, which demonstrated that rhPTX-2 is generally well tolerated over 128 weeks in patients with IPF, with no new safety signals observed vs the randomized period [13].

rhPTX-2 is a recombinant form of human pentraxin-2, an endogenous protein that plays a critical beneficial role in fibrosis [17–20]. Plasma levels of pentraxin-2 have been found to be lower in patients with IPF compared with healthy age-matched controls [19]. Like endogenous pentraxin-2, rhPTX-2 shifts the balance of monocyte differentiation to inhibit pro-inflammatory, pro-fibrotic macrophages, and fibrocytes, and to induce regulatory macrophages [19–21]. This represents a different mechanism of action compared with existing antifibrotic treatments for IPF, which combined with data from preclinical studies [19–21] and a Phase I trial [22], suggests that rhPTX-2 has therapeutic potential in IPF. PRM-151-202 was designed to investigate this therapeutic potential further in a Phase II clinical trial setting.

In the 28-week, randomized, placebo-controlled period of PRM-151-202, rhPTX-2 was generally well tolerated in patients with IPF [13]. The majority of TEAEs were mild or moderate in severity, and among the 9% of patients who experienced serious TEAEs, no events were considered related to rhPTX-2 treatment. The most common TEAEs with rhPTX-2 were cough, fatigue, nasopharyngitis, headache, and IPF.

Interim analyses of the OLE data, up to Week 76, were consistent with the randomized period safety profile [15]. The most frequently reported TEAEs were IPF-related symptoms or upper respiratory tract infections. Additionally, the types and frequencies of TEAEs between patients who started rhPTX-2 and patients who continued rhPTX-2 in the OLE did not differ greatly. These findings are consistent with the results of the entire OLE study, which demonstrated that rhPTX-2 was generally well tolerated up to Week 128, with no new safety signals observed vs the randomized period [13]. As in the randomized period, most TEAEs were considered mild or moderate, and the majority of serious TEAEs were not considered to be related to treatment with rhPTX-2. The TEAE profile was also consistent with the randomized period and the expected profile of events in this study population, with the most commonly reported TEAEs found to be IPF, dyspnea, cough, upper respiratory tract infection, and fatigue. Differences of ≥ 10% between the two patient groups at Week 128 were seen for dyspnea and diarrhea (more frequent in patients who continued rhPTX-2), and bronchitis and cough (more frequent in patients who started rhPTX-2). The clinical relevance of these differences is difficult to assess because all patients were receiving rhPTX-2 during the OLE. Although there may have been potential selection bias for patients who could tolerate rhPTX-2 in the subgroup who continued rhPTX-2, this is likely to have had a minimal effect since few patients discontinued before entering the OLE, with an equal proportion of patients who received rhPTX-2 or placebo in the double-blind phase discontinuing due to TEAEs. In addition, although there was no difference in the rate of background antifibrotic therapy use at Week 128, these events may be associated with the underlying IPF or background treatment. The frequency of TEAEs leading to treatment discontinuation – most commonly being IPF (9%) – may be a function of the progressive nature of IPF and the long duration of follow-up. It is also important to note that the overall incidence of patients with any TEAE was similar to that reported in RECAP, a long-term OLE study of pirfenidone in patients with IPF, where the median treatment duration was 88 weeks [23]. Moreover, the proportion of patients who discontinued early in this Phase II OLE study due to AEs was lower than that reported in RECAP (16.5% vs 33.8%, respectively) [23].

The majority of IRRs (reported in 10 patients during the OLE) were mild to moderate with no need for medical intervention. Although the most common IRR was blood pressure fluctuation, these events were not suggestive of being immune-mediated; indeed, vital sign measurements (before and after dosing, and every 15 min during infusion) recorded no appreciable change in blood pressure overall during the OLE, so the IRR reports may reflect the natural variability of frequent monitoring. Moreover, infusion duration of greater than or less than 1 h were largely similar between groups across the OLE. These results suggest that rhPTX-2 is well tolerated in patients with IPF over 128 weeks.

In addition to safety, the efficacy of rhPTX-2 in patients with IPF was also assessed in PRM-151-202. In the randomized period of the study, for the primary endpoint of least squares mean change from baseline in percent predicted FVC at Week 28, rhPTX-2 showed a significant benefit vs placebo (− 2.5% vs − 4.8%, respectively; *p* = 0.001) [13]. Decline in 6MWD at Week 28 was also significantly lower in the rhPTX-2 arm vs the placebo arm (− 0.5 m vs − 31.8 m, respectively; *p* < 0.001). There were no significant treatment differences in changes from baseline in percent predicted DLco. Efficacy endpoints evaluated up to Week 52 in the interim OLE analyses suggested a persistent treatment benefit of rhPTX-2 on FVC and 6MWD in patients who were initiated on rhPTX-2 at baseline of the randomized period and continued treatment in the OLE period. Among patients who were randomized to placebo and initiated rhPTX-2 at the beginning of the OLE at Week 28, a treatment benefit of rhPTX-2 was demonstrated through a reduction in the estimated rates of FVC and 6MWD decline vs the randomized period [13, 15]. Looking at efficacy over 128 weeks, the observed data suggest that there may be a trend towards a treatment effect of rhPTX-2 on FVC and 6MWD; however, it is not possible to draw any definitive conclusions from these data due to reduced patient numbers, increased variability of data at later time points, and the lack of a comparator group. There may also be a trend towards a lower rate of decline in lung function in patients who continued treatment with rhPTX-2 across both study phases, although this could have arisen from factors other than previous placebo use.

Based on the safety and efficacy results demonstrated during PRM-151-202, further investigation of rhPTX-2 in patients with IPF is warranted. STARSCAPE (ClinicalTrials.gov: NCT04552899) is a randomized, double-blind, placebo-controlled, international Phase III study designed to evaluate the efficacy and safety of rhPTX-2 in patients with IPF over 52 weeks. An OLE study (ClinicalTrials.gov: NCT04594707) exploring the long-term safety, efficacy, and pharmacokinetics of rhPTX-2 will also be conducted. Patients from both the randomized Phase III study and the Phase II PRM-151-202 trial (ClinicalTrials.gov: NCT02550873) will be invited to participate.

There are limitations in the available data for analysis of the OLE. Demographic and clinical characteristics were not re-collected at the beginning of the OLE period, although some data on clinical characteristics were collected at the end of the randomized period. In addition, the clinical relevance of the differences in the TEAE profile during the OLE period between patients originally randomized to rhPTX-2 and those randomized to placebo are difficult to interpret due to lack of a comparator group, with all patients receiving rhPTX-2 during the OLE period. Finally, there was withdrawal of a large proportion of patients prior to Week 128, which restricts interpretation of the findings and means that definitive conclusions cannot be made regarding the clinical outcomes. Study withdrawal was multifactorial, and reasons included disease progression and patient request. Of the patients who provided specific reasons for requesting to withdraw from the study (n = 18/19), no patient indicated withdrawal due to safety or tolerability issues. Travel to the study site to receive IV treatment every 4 weeks was required, thus, travel fatigue or hardship played a role in choosing to withdraw from the study. It is relevant to note that the study was completed in April 2019, therefore behavior patterns were not influenced by the COVID-19 pandemic.

## Conclusions

In summary, the efficacy and safety of rhPTX-2 in patients with IPF was investigated during an extended duration of 128 weeks in study PRM-151-202. rhPTX-2 was generally well tolerated in the long term, with similar TEAE profiles observed as those in the randomized period (13). No new safety signals were observed in the OLE period compared with the randomized period. The observed efficacy data up to Week 128 were suggestive of a trend towards a treatment effect of rhPTX-2. However, the limitations of the data prevent any definitive conclusions concerning efficacy. To assess the clinical significance, these findings will be investigated further in a 52-week Phase III study of rhPTX-2 in patients with IPF (STARSCAPE) that will be followed by another long-term OLE study. If Phase III study results mirror those of Phase II, rhPTX-2 holds great promise as add-on therapy to existing antifibrotics to further slow progression of IPF, and also as effective monotherapy for patients intolerant of current therapeutic options.

## Data Availability

Qualified researchers may request access to individual patient-level data through the clinical study data request platform (https://vivli.org/). Further details on Roche’s criteria for eligible studies are available here (https://vivli.org/members/ourmembers/). For further details on Roche’s Global Policy on the Sharing of Clinical Information and how to request access to related clinical study documents, see here (https://www.roche.com/research_and_development/who_we_are_how_we_work/clinical_trials/our_commitment_to_data_sharing.htm).

## Abbreviations

6MWD: 6-minute walk distance
ADA: Anti-drug antibody
AE: Adverse event
ATS: All Treated Set
ATS-OLE: All Treated Set of the open-label extension
CI: Confidence interval
DLco: Carbon monoxide diffusing capacity
FEV_1_: Forced expiratory volume in 1 second
FVC: Forced vital capacity
IPF: Idiopathic pulmonary fibrosis
IRR: Infusion-related reaction
IV: Intravenous
K-BILD: King’s Brief Interstitial Lung Disease questionnaire
LCQ: Leicester Cough Questionnaire
OLE: Open-label extension
rhPTX-2: Recombinant human pentraxin-2
SAF: Safety Analysis Set
SAF-OLE: Safety Analysis Set of the open-label extension
SD: Standard deviation
TEAE: Treatment-emergent adverse event
